# State anxiety and information processing: A 7.5% carbon dioxide challenge study

**DOI:** 10.3758/s13423-017-1413-6

**Published:** 2018-02-01

**Authors:** Kayleigh E. Easey, Jon C. Catling, Christopher Kent, Coral Crouch, Sam Jackson, Marcus R. Munafò, Angela S. Attwood

**Affiliations:** 1 MRC Integrative Epidemiology UnitUniversity of Bristol, Bristol, UK; 2 UK Centre for Tobacco and Alcohol Studies, University of Bristol, Bristol, UK; 3 School of Experimental Psychology, University of Bristol, 12a Priory Road, Bristol, BS8 1TU UK; 4 School of Psychology, University of Birmingham, Birmingham, UK

**Keywords:** Anxiety, Visual perception, Auditory perception, Human factors

## Abstract

We used the 7.5% carbon dioxide model of anxiety induction to investigate the effects of state anxiety on simple information processing. In both high- and low-anxious states, participants (*n* = 36) completed an auditory–visual matching task and a visual binary categorization task. The stimuli were either degraded or clear, so as to investigate whether the effects of anxiety are greater when signal clarity is compromised. Accuracy in the matching task was lower during CO_2_ inhalation and for degraded stimuli. In the categorization task, response times and indecision (measured using mouse trajectories) were greater during CO_2_ inhalation and for degraded stimuli. For most measures, we found no evidence of Gas × Clarity interactions. These data indicate that state anxiety negatively impacts simple information processing and do not support claims that anxiety may benefit performance in low-cognitively-demanding tasks. These findings have important implications for understanding the impact of state anxiety in real-world situations.

Anxiety is a common feature of everyday life. From an evolutionary perspective, state anxiety may have adaptive value because it can focus attention to salient stimuli, particularly those that signal threat. However, “fight or flight” reactions are often inappropriate in modern situations, which require controlled and reasoned cognitive processing. For example, in many professions, including medicine, aviation, and the military, workers are required to process information, make reasoned decisions, and perform intricate skills in states of heightened anxiety. Impairments in any of these processes can have disastrous consequences. Indeed, it is estimated that the majority of fatal aviation accidents can be linked to human error (International Air Transport Association, 2013). Understanding how anxiety affects these abilities, and how we may attenuate impairments, is an important area of research with wide-reaching implications.

High trait anxiety has been associated with poorer outcomes on a number of cognitive parameters, in both animals (Herrero, Sandi, & Venero, 2006) and humans (Byron & Khazanchi, 2011; Gawda & Szepietowska, 2016). Traditionally, anxiety has been associated with a hypersensitivity to threat (Spielberger, 1972). However, more recently, Bishop (2009) suggested that a broader dysregulation of the attentional control system underlies more generic impairments in cognitive performance in high trait anxiety.

There is increasing evidence that *state* anxiety may have similar negative effects on cognition. State anxiety is a fundamental component of the anxiety profile, since its frequency and severity are heightened in trait-anxious individuals. Furthermore, anxious states are not restricted to “high trait” subgroups. In numerous situations, individuals have to perform in anxiogenic conditions, even though skills are routinely trained and practiced in nonanxious states. Because these situations often involve complex tasks, the extent to which anxiety negatively impacts on different cognitive processes is often unclear. It is plausible that under some circumstances, heightened state anxiety might benefit performance, particularly when cognitive load is low (Diaper et al., 2012).

In the present study, we investigated the effects of state anxiety on simple auditory and visual information processing. Because real-world telecommunication devices are prone to interference that degrades signal quality, and visual stimuli may be viewed in suboptimal conditions (e.g., blurred CCTV), we included both clear and degraded stimuli in both tasks. We used the 20-min 7.5% CO_2_ inhalation challenge (Bailey, Argyropoulos, Kendrick, & Nutt, 2005) to induce state anxiety. This model has produced reliable increases in state anxiety in a number of studies (Attwood, Catling, Kwong, & Munafò, 2015; Attwood et al., 2017; Attwood, Penton-Voak, Burton, & Munafò, 2013; Button, Lewis, Penton-Voak, & Munafò, 2013; Garner, Attwood, Baldwin, James, & Munafò, 2011), and although there is evidence that its effects might be somewhat larger in individuals prone to anxiety (Fluharty, Attwood, & Munafò, 2016), in our experience nonresponders are rare. We hypothesized that performance would be poorer during 7.5% CO_2_ inhalation (as compared to inhalation of air) and in the degraded (as compared to the clear) condition, and that the magnitude of the degradation effect would be greater for CO_2_ than for air inhalation.

## Method

The protocol for this study was preregistered on the Open Science Framework: https://osf.io/gqs93/. Ethics approval was obtained from the Faculty of Science Research Ethics Committee at the University of Bristol (reference: 30101413461).

### Participants

Thirty-six healthy volunteers (23 female, 13 male) were recruited from among members of the University of Bristol and the local community. The exclusion criteria included being under 18 or over 50 years of age, daily smoking, history of drug/alcohol dependency, pregnancy or breast feeding, recent use of prescribed or illicit drugs, uncorrected visual or hearing problems, diagnosed medical illness, and not being registered with a general practitioner. Pregnancy and recent drug use were assessed by urine screen, whereas all other criteria were confirmed by self-report. Participants were also excluded if they had high systolic or diastolic blood pressure (SBP/DBP) (<140/90 mmHg), bradycardia or tachycardia (<50 or >90 beats per minute), or a body mass index (BMI) outside a healthy range (<18 or >30 kg/m^2^) (all physically assessed). Psychiatric health was assessed using a truncated MINI-International Neuropsychiatric Interview (Sheehan et al., 1998). Participants refrained from consuming alcohol for 36 h prior to the study day. Expired breath alcohol and carbon monoxide readings were taken, and participants were excluded if the readings were >0 or ≥10, respectively. To achieve our target of 36 participants, a total of 45 individuals were screened. The nine participants who were not enrolled were deemed ineligible because they had either failed to meet one or more of the eligibility criteria or did not respond to further contact.

The sample size was determined from the effect sizes obtained in a previous study investigating the effects of CO_2_ inhalation on speech perception (Mattys, Seymour, Attwood, & Munafò, 2013). This study indicated an effect size of *dz* = 0.57 (difference in phoneme recognition during 7.5% CO_2_ inhalation vs. air inhalation). On the basis of these data, we required a sample size of *n* = 36 to achieve 90% power at an alpha level of 5%.

### Design

Both gas (air, 7.5% CO_2_) and stimulus clarity (clear, degraded) were manipulated within subjects. The gas and task orders were counterbalanced across participants.

### Materials

The gases were either 7.5% CO_2_/21% oxygen/71.5% nitrogen or medical air (21% oxygen; BOC Ltd.). These were administered using an oro-nasal mask (Hans Rudolph, Kansas City, MO, USA). For safety reasons, the gas was administered single blind. Questionnaires^1^ included the Spielberger State–Trait Anxiety Inventory (state and trait; Spielberger, Gorsuch, Lushene, Vagg, & Jacobs, 1983), Positive and Negative Affect Schedule (PANAS; Watson, Clark, & Tellegen, 1988), Anxiety Sensitivity Index (ASI) (Derryberry & Reed, 2002), and Eysenck Personality Questionnaire Revised (EPQR-R) (Eysenck & Eysenck, 1991).

The computer tasks were displayed on a 17-in. LCD monitor at a resolution of 1,280 × 1,024 with a 60-Hz refresh rate. Each task was completed twice (once during each inhalation).

### Audio–visual matching task

On each trial, a black-and-white image was presented centrally on screen and a verbal descriptor was played simultaneously through headphones. On 50% of trials the image matched the verbal descriptor. Participants were required to indicate whether the descriptor matched the image by pressing designated keys on the keyboard. If a response was not made within 4,000 ms, the trial was terminated. On 50% of the trials the descriptor was clear, and on 50% it was degraded (i.e., muffled). The audio clips were degraded using low-pass filtering with a 1,000-Hz cutoff and a 32-dB/octave roll-off, resulting in them sounding muffled. Therefore, four conditions were presented (clear/matching, degraded/matching, clear/nonmatching, degraded/nonmatching), each comprising 50 trials. The outcome variables were reaction time (in milliseconds; correct responses only), hit rate (i.e., number of correct responses in matching trials), and false alarm rate (i.e., number of errors made on nonmatched trials, in which participants reported that the visual and auditory stimuli matched when the stimuli did not).

### Visual binary categorization task

**Fig. 1:**
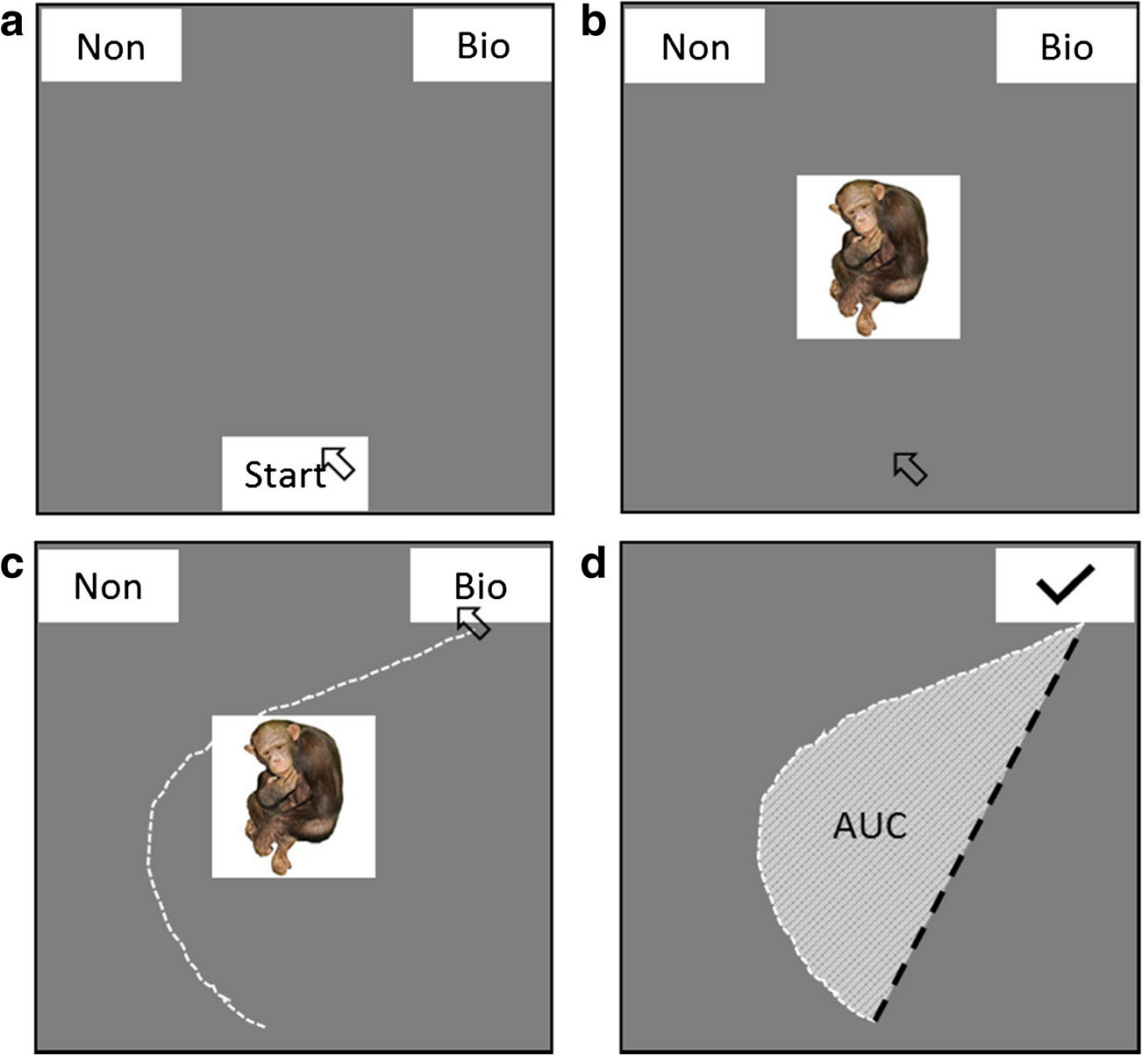
Structure of a trial (A–C) for the binary categorization task. (A) Participants initiated the trial by clicking the “Start” button. (B) An image (clear, in the example) was displayed. (C) Participants moved the mouse cursor (broken white line, not visible on trial) and clicked on a response option. (D) Area under the curve (AUC; i.e., the filled area) is the area between the actual trajectory (broken white line) and an idealized trajectory (broken black line, drawn from the starting *x*, *y* position to the finishing *x*, *y* position of the actual trajectory). Note that the images are not to scale.

A schematic of the task is shown in Fig. 1. On each trial, a pictorial stimulus appeared centrally on the screen. Participants were required to identify whether the image was biological or nonbiological. The labels “Bio” and “Non” appeared in response boxes in the top right and left of the screen (positions counterbalanced across participants). Participants responded using a standard USB mouse.

The stimuli consisted of 160 color 190 × 190 pixels (5.72 × 5.72 deg of visual angle) images taken from the Bank of Standardized Stimuli (Brodeur, Dionne-Dostie, Montreuil, & Lepage, 2010), 50% of which were biological items and 50% nonbiological items. Furthermore, 50% of the images in each category were degraded using a 50% Gaussian blur. Category type (biological or nonbiological) and image type (clear or degraded) were randomly intermixed.

Each trial was initiated by clicking within a “Start” box located at the bottom of the screen. A randomly selected stimulus was then presented, and participants were instructed to respond as quickly and as accurately as possible. To encourage online decision making, a prompt appeared at end of any trial on which participants failed to move the mouse within 1,000 ms of stimulus onset. There was a 500-ms intertrial interval, and the task consisted of 80 trials. The stimulus presentation and response collection were achieved via MouseTracker software (Freeman & Ambady, 2010), which has been used in previous categorization studies (Dale, Kehoe, & Spivey, 2007).

The outcome variables for this task comprised: (1) reaction time (correct responses), measured from the point at which the “Start” button was clicked to the point at which the participant clicked on either the “Bio” or “Non” response box (i.e., until the response was terminated), and (2) average area under the curve (AUC), which is a measure of attraction to the incorrect response and therefore gives an indication of indecision (with higher AUCs representing greater indecision; see panel D of Fig. 1). Before AUC was calculated, screen coordinates were transformed into a standard coordinate space from (– 1, 1.5; top left) to (1, 0; bottom right); all response trajectories to the left response were flipped onto the right response (allowing us to compare the biological and nonbiological images). We also time normalized all trajectories via linear interpolation into 100 time bins, to allow us to compare the spatial trajectories of responses with different reaction times (see Freeman & Ambady, 2010, for fuller discussion of the analysis techniques).

### Procedure

Prior to the session, a telephone screen assessed basic eligibility. Eligible participants attended a single test session, at which full written informed consent was obtained and further screening assessments were conducted. If eligibility was met, baseline questionnaire (STAI, SSAI, PANAS, and ASI) and cardiovascular (blood pressure [BP] and heart rate [HR]) measures were recorded. The inhalation began with 60 s of free breathing before the tasks were started (this allowed for the gas to start taking effect before data collection began). Inhalations then continued for the duration of the two computer tasks (up to 20 min for each inhalation). Immediately after each inhalation, measures of BP, HR, SSAI, and PANAS were completed, and there was a 30-min washout period between inhalations. The second inhalation followed the same procedure as the first. After the inhalations were complete, participants remained in the room for a minimum of 20 min, to allow any effects to dissipate, and completed the EPQ-r. Participants were then debriefed and reimbursed. A follow-up call was conducted 24 h later to assess whether any adverse events had occurred.

## Results

The data that form the basis of the results presented here are available from the University of Bristol Research Data Repository (http://data.bris.ac.uk/data/), (10.5523/bris.1bvk8fmcvv4m1b6c1p5efsgvr).

Task data were assessed for normality, and where deviations were observed, appropriate transformations were run. For ease of interpretation, untransformed data and analyses are presented where the results did not differ after transformation.

### Characteristics of participants

The participants (n = 36; 39% male) were between 19 and 49 years of age (M = 22, SD = 5). Their body mass indexes ranged between 18 and 29 (M = 23, SD = 3). In terms of test results, the participants’ STAI Trait and ASI scores ranged between 22 and 46 (M = 32, SD = 7) and between 2 and 31 (M = 15, SD = 6), respectively. Their EPQ-R scores ranged between 0 and 13 (M = 6, SD = 3) for psychoticism, 2 and 21 (M = 9, SD = 4) for neuroticism, and 8 and 23 (M = 17, SD = 4) for extraversion.

### Subjective and cardiovascular effects

PANAS-negative data in the air condition were positively skewed due to two outlying scores, which were likely due to high anticipatory anxiety in the air condition. Removing these data points increased the comparison effect size but did not change the overall effect, and therefore the data from the full sample are reported. State anxiety (STAI), negative affect (PANAS-negative), SBP, DBP, and HR were higher, and positive affect (PANAS-positive) was lower, after CO_2_ than after air inhalation (see Table 1), confirming the validity of the anxiety manipulation.

**Table 1:** State anxiety, affect and cardiovascular *t* test comparison data

	Mean Difference (*SD*): Air vs. CO_2_	Delta % CO_2_	Delta % Air	Effect Size (*dz)*	*df*	95% CI	*p* Value
STAI State	20.8 (10.7)	97.3	20.4	1.95	35	17.2 to 24.4	<.001
PANAS-pos.	– 6.6 (6.4)	– 35.8	– 17.1	1.03	35	– 8.7 to – 4.4	<.001
PANAS-neg.	9.9 (7.1)	97.7	7.8	1.40	35	7.5 to 12.3	<.001
SBP	13.4 (12.6)	13.3	0.4	1.06	35	9.1 to 17.7	<.001
DBP	5.0 (12.8)	10.9	3.4	0.40	35	0.7 to 9.3	.025
HR	16.3 (18.5)	25.8	1.8	0.88	35	10.0 to 22.5	<.001

STAI, Spielberger State–Trait Anxiety Inventory; PANAS, Positive and Negative Affect Schedule; SBP, systolic blood pressure; DBP, diastolic blood pressure; HR, heart rate. Delta scores are derived from the group means.

### Audio–visual task

For accuracy, hit rate and false alarm rate were calculated separately for the clear and degraded conditions. The mean reaction time (in milliseconds) was calculated for correct responses (matching trials only). The total hit data were negatively skewed, and log_10_ transformations were used to correct this (Tabachnick & Fidell, 2007).

**Fig. 2:**
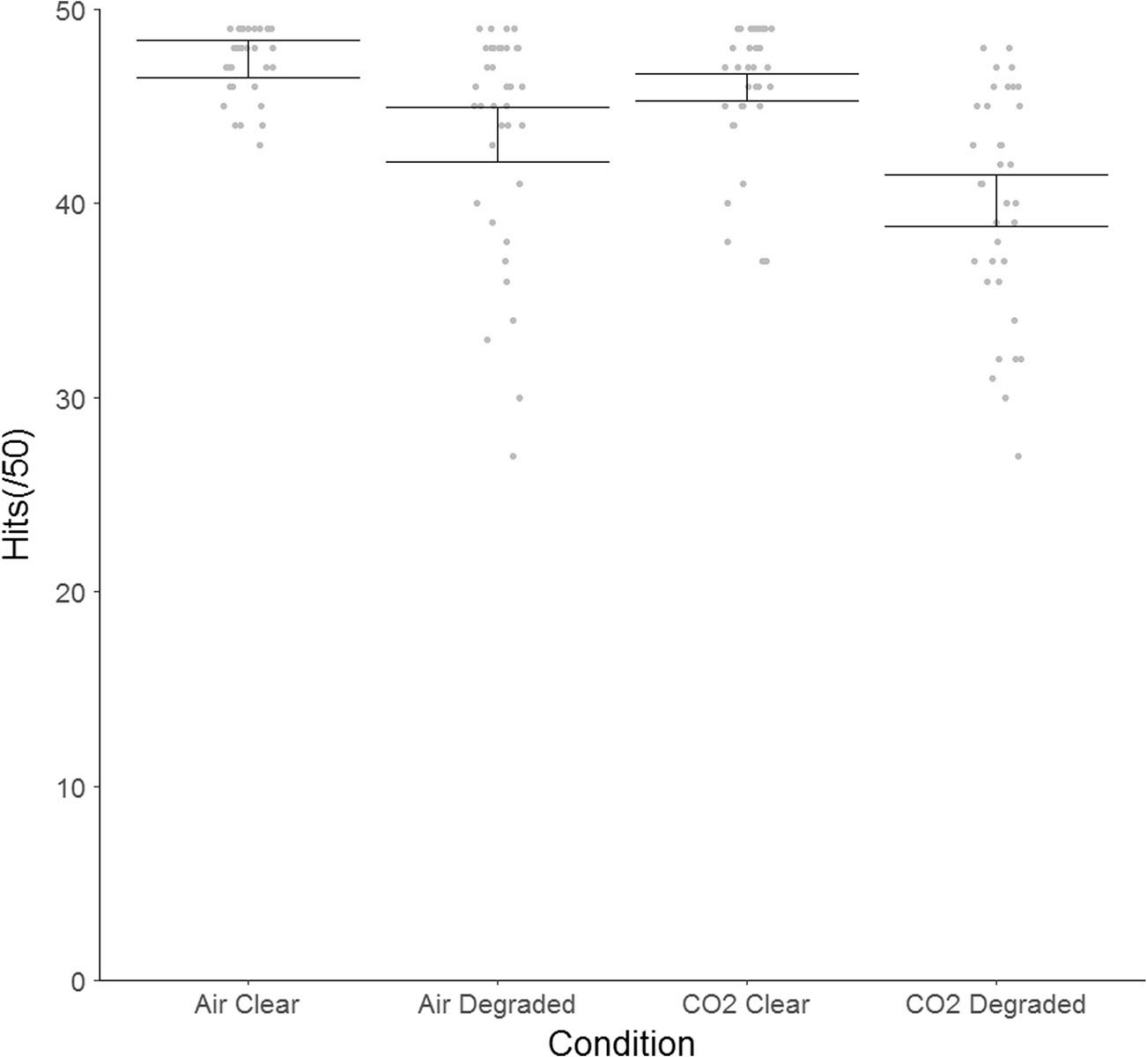
Mean hits (out of 50) in the clear and degraded conditions during CO_2_ and air inhalation (untransformed data). Circles are the individual data points, and horizontal lines represent within-subjects confidence intervals, calculated using Morey (2008).

Using untransformed data, we observed a main effect of gas on hit rates [*F*(1, 35) = 18.50, *p* < .001, *η*_p_^2^ = .35], indicating more hits in the air than in the CO_2_ condition. We also found a main effect of clarity [*F*(1, 35) = 69.42, *p* < .001, *η*_p_^2^ = .67], indicating greater accuracy when the verbal descriptor was clear than when it was degraded (see Fig. 2). There was weak evidence of a Gas × Clarity interaction [*F*(1, 35) = 3.32, *p* = .077, *η*_p_^2^ = .09]. Post-hoc *t* tests stratified by gas revealed that although there were fewer hits in the degraded than in the clear condition during both air (*t* = 4.46, *df* = 35, *p* < .001, *dz* = 0.73, 95% CI: 2.10 to 5.62) and CO_2_ (*t* = 8.27, *df* = 35, *p* = < .001, *dz* = 1.38, 95% CI: 4.38 to 7.23) inhalation, the magnitude of this effect was greater in the CO_2_ condition (as indicated by a greater effect size). This interaction was no longer observed once the data were transformed [*F*(1, 35) = 0.19, *p >* .250, *η*_p_^2^ = .005].

For false alarms, one outlier was observed and removed from the analysis. There was evidence of main effects of both gas [*F*(1, 34) = 10.45, *p* = .003, *η*_p_^2^ = .24] and clarity [*F*(1, 34) = 15.05, *p* < .001, *η*_p_^2^ = .31], with more false alarms during CO_2_ (*M* = 2.2, *SD* = 2.2) than during air (*M* = 1.1, *SD* = 1.0) inhalation, and in the degraded (*M* = 2.1, *SD* = 1.6) than in the clear (*M* = 1.2, *SD* = 1.6) conditions. We found no evidence of a Gas × Clarity interaction (*p* = .82).

There was evidence of a positive skew in mean reaction times. The findings did not differ using the transformed data, and therefore only statistics from the untransformed analysis are presented. Main effects of both gas [*F*(1, 35) = 5.93, *p* = .020, *η*_p_^2^ = .15] and clarity [*F*(1, 35) = 57.88, *p* < .001, *η*_p_^2^ = .63] emerged, with slower reaction times during CO_2_ inhalation (*M* = 908 ms *SD* = 204 ms) than during air inhalation (*M* = 840 ms, *SD* = 171 ms), and in degraded (*M* = 916 ms, *SD* = 170 ms) than in clear (*M* = 833 ms, *SD* = 173 ms) conditions. There was no evidence of a Gas × Clarity interaction (*p* > .250).

### Visual binary categorization task

Only correct responses were analyzed (96% of trials). Responses that were initiated after 1,000 ms were discarded (1% of trials).

#### Reaction time

A 2 Gas (CO_2_, air) × 2 Clarity (clear, degraded) repeated measures analysis of variance (ANOVA) was conducted on correct reaction times. We found evidence of main effects of gas [*F*(1, 35) = 6.43, *p* = .016, *η*_p_^2^ = .16] and clarity [*F*(1, 35) = 35.37, *p* < .001, *η*_p_^2^ = .50]. Reaction times were slower during CO_2_ (*M* = 1,411 ms, *SD* = 338 ms) than during air (*M* = 1,279 ms, *SD* = 202 ms) inhalation, and for degraded (*M* = 1,408 ms, *SD* = 317 ms) than for clear (*M* = 1,282 ms, *SD* = 235 ms) images. There was no clear evidence of a Gas × Clarity interaction (*p* = .125), and no evidence of main effects or interactions for the average time to move the mouse (*F*s < 0.34), suggesting that participants were trying to move the mouse as soon as the stimulus appeared across all conditions.

#### Area under the curve

Density plots of the pooled AUC data did not show evidence of bimodality (Hartigan’s *D* = 0.002, *p* = .999). We therefore conducted a 2 Gas (CO_2_, air) × 2 Clarity (clear, degraded) repeated measures ANOVA on the average AUC values. The AUC values were higher after CO_2_ (*M* = 0.68, *SD* = 0.44) than after air (*M* = 0.57, *SD* = 0.38) inhalation [*F*(1, 35) = 15.03 *p* < .001, *η*_p_^2^ = .30], suggesting greater indecision when anxious. The AUC values were also higher for the degraded images (*M* = 0.67, *SD* = 0.42) than for the clear images (*M* = 0.58, *SD* = 0.41) [*F*(1, 35) = 5.99 *p* = .020, *η*_p_^2^ = .15], suggesting that participants were more indecisive when the images were degraded. There was no evidence of an interaction between gas and clarity (*p* = .97).

## Discussion

We found evidence that state anxiety impairs information processing during simple cognitive tasks. These impairments consisted of fewer hits and more false alarms in the auditory–visual task and slower reaction times and greater indecision in the visual categorization task. These data support our hypothesis that performance suffers when participants are in an anxious state.

In contrast, the data did not support the hypothesis that the detrimental effect of stimulus degradation would be greater during CO_2_ inhalation. Other possible explanations of this lack of interaction between gas and stimulus degradation may be that the mechanism by which anxiety affects simple processing is different from the processes involved in differentiating clear and degraded stimuli. That is, the two main effects may have operated at different levels of cognition. For both tasks, performance was worse when the stimuli were degraded, indicating that the degradation manipulation was successful. However, it may be that the degree of difference in difficulty was not large enough to detect reliable differences between the gas inhalations, and effects of difficulty might be more apparent with more complex tasks. Observation of the means, particularly the hit data in the auditory–visual task, indicated that degradation effects were greater with CO_2_ but that this effect was not robust enough to be supported statistically. It is plausible that there was a small interaction effect that this study was not sufficiently powered to detect, and this warrants further investigation.

These findings are particularly noteworthy because the tasks required very simple information processing. Previous studies on attentional processing have indicated that the negative influence of anxiety may be limited to complex tasks, and that there may be some benefit of acute anxiety when tasks are simple (Diaper et al., 2012). Our data indicate that other cognitive processes, such as information processing and simple decision making, are negatively affected by state anxiety even when cognitive demand is low. It is plausible that processing advantages of state anxiety might arise when there is a need to process threat-related information. In support of this conjecture, Garner et al. (2011) reported difficulties in disengaging from threatening information during 7.5% CO_2_ inhalation. Although this was interpreted as impaired performance (since the aim of the task was to focus on nonthreat stimuli), this implies that there might be a benefit of anxiety if the aim of the task is to focus attention on threat information. However, our findings suggest that in situations in which this is not the primary aim, state anxiety negatively affects performance even in very simple tasks.

These findings have particular importance for real-world applications, where information processing and decision making are required in stressful conditions. For example, disruption to the processing of simple auditory information through communication systems such as two-way radios could have serious consequences for professionals involved in maintaining the safety of others (e.g., communication between pilots and air traffic control). This emphasizes a need for professional training paradigms to take account of these processing impairments, to mitigate their impact when skills have to be applied outside of the training contexts.

Future research could extend these findings by increasing task complexity in order to investigate whether gas–clarity interactions are more robust when the tasks are more cognitively demanding. The importance of this work is exemplified by the recent emergence of cognitive-training paradigms. The tractable nature of these processes mean that CO_2_ inhalation could be paired with training in order to improve anxiety-related performance.

### Author note

A.S.A. and M.R.M. are members of the UK Centre for Tobacco and Alcohol Studies. Funding from the British Heart Foundation, Cancer Research UK, Economic and Social Research Council, Medical Research Council, and National Institute for Health Research, under the auspices of the UK Clinical Research Collaboration, is gratefully acknowledged. This study was supported in part by the Medical Research Council and the University of Bristol (Grant No. MC_UU_12013/6). A.S.A., J.C.C., and C.K. developed the study concepts. All authors contributed to the study design and protocol development. A.S.A., J.C.C., C.K., and S.J. developed the study materials (including the computer tasks and stimuli). The testing and data collection were conducted by S.J. and C.C. Finally, K.E.E., C.C., S.J., C.K., and A.S.A. performed the data analyses, and A.S.A. and K.E.E. drafted the manuscript. All authors contributed to interpretation of the results and critical review of the manuscript. All authors approved the final version of the manuscript for submission. A.S.A. and M.R.M. have acted as consultants for pharmaceutical companies on use of hypercapnic challenges in psychopharmacological research. The authors have no other conflicts of interest.

## Footnotes

1 Full details of the sample means and standard deviations are available upon request.
